# Optimization of Ultrasound-Assisted Extraction of Flavonoids from Olive (*Olea europaea*) Leaves, and Evaluation of Their Antioxidant and Anticancer Activities

**DOI:** 10.3390/molecules23102513

**Published:** 2018-09-30

**Authors:** Bixia Wang, Jipeng Qu, Siyuan Luo, Shiling Feng, Tian Li, Ming Yuan, Yan Huang, Jinqiu Liao, Ruiwu Yang, Chunbang Ding

**Affiliations:** 1 College of Life Sciences, Sichuan Agricultural University, Yaan 625014, China; 696wbx@163.com (B.W.); ququ8312@163.com (J.Q.); luosiyuan1998@163.com (S.L.); fengshilin@outlook.com (S.F.); lit@sicau.edu.cn (T.L.); yuanming@sicau.edu.cn (M.Y.); shirley11hy@163.com (Y.H.); liaojinqiu630@sicau.edu.cn (J.L.); yrwu@sicau.edu.cn (R.Y.); 2 College of Environmental Science and Engineering, China West Normal University, Nanchong 637009, China; 3 College of Agricultural Science, Xichang University, Xichang 615000, China

**Keywords:** olive leaves, flavonoids, ultrasound-assisted extractio, antioxidant activity, anticancer activity

## Abstract

*Olea europaea* leaves are the major byproduct of olive farming. In this study, ultrasound-assisted extraction of flavonoids from olive leaves was optimized using response surface methodology, and the flavonoid compounds and their antioxidant and anticancer activities were investigated by high performance liquid chromatography. The results showed that the optimized conditions for achieving the maximum yield of flavonoids (74.95 mg RE/g dm) were 50 °C temperature, 270 W power, 50 min time, and 41 mL/g liquid-solid ratio. There was a significant difference in the total flavonoid content between the aged and young leaves harvested in April and July, and six main components were quantified. Among them, luteolin-4’-*O*-glucoside was the most predominant flavonoid compound, followed by apigenin-7-*O*-glucoside and rutin. Olive leaves also contained small amounts of luteolin, apigenin, and quercetin. Additionally, excellent antioxidant activity was exhibited when tested with the DPPH assay; superoxide radical-scavenging ability and reducing power was also tested. The anticancer activity of the flavonoids was assessed using HeLa cervical cancer cells, and it was observed that increasing concentrations of olive leaf flavonoids resulted in decreased cancer cell viability. These results suggest that the flavonoids from olive leaves could be used as a potential source of natural antioxidants for the pharmaceutical and food industries.

## 1. Introduction

*Olea europaea* L., an evergreen tree belonging to the *Oleaceae* family, is widely distributed in many Mediterranean countries [1]. Olive trees were introduced to China in 1956 as an important economic crop, and thereafter, they were widely cultivated along the Jinsha River in the hot, arid valley [2]. A huge quantity of olive leaves is generated as an agricultural byproduct of the olive oil industry and accumulates during pruning [3]. According to statistics, almost 600,000 tons of olive leaves are produced annually in China [4]. However, these byproducts are burned or directly discarded, causing environmental pollution and waste of a resource.

Recent studies have demonstrated that olive leaves contain a wide range of bioactive components including hydroxycinnamic acid derivatives, flavonoids, secoiridoids, and triterpenes [5], and the leaves have been used as a folk remedy to treat influenza, malaria, diarrhoea, and surgical infections [6]. Flavonoids are one of the most important compounds in olive leaves [7], possessing cardioprotective, antihypertensive, antibacterial, anti-inflammatory, and antioxidant health-promoting properties [8,9,10]. The anticancer activity was investigated by Milanizadeh, et al. [11], who demonstrated that olive leaf extract reduced breast cancer cell volume and weight in mice. Due to their various biological activities, olive leaves have been widely applied in the medicinal, food, and cosmetic industries [12].

Conventional extraction procedures of flavonoids from plant materials, such as boiling, and refluxing and heating, usually require much time, energy, and non-recoverable chemicals and solvents [13]. Ultrasound-assisted extraction (UAE) is an ideal extraction method capable of producing large quantities of flavonoids with inexpensive, simple, and efficient techniques [14]. Recently, response surface methodology (RSM), a mathematical and statistical tool, has been successfully applied in the optimization of UAE processes [15]. To the best of our knowledge, there are few studies on the extraction of flavonoids from olive leaves [16].

In this study, RSM was employed to optimize UAE parameters including temperature, time, power, and liquid-solid ratio for flavonoid extraction from olive leaves. Then, the amounts of flavonoid compounds were measured between the aged and young leaves harvested in April and July, respectively. Additionally, we also evaluated the antioxidant and anticancer activities of flavonoids in vitro.

## 2. Results and Discussion

### 2.1. Single Factor Experiment

#### 2.1.1. Effect of Extraction Temperature

**Figure 1:**
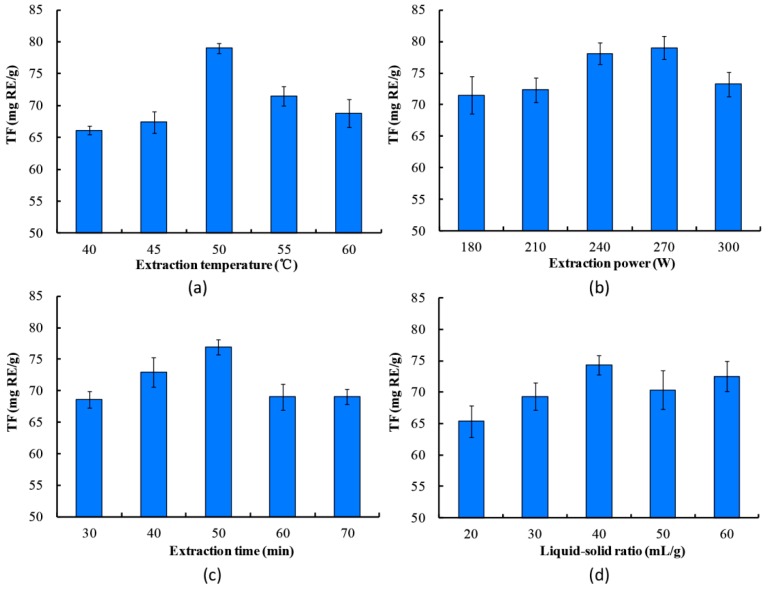
Single-factor effect on the total flavonoids (TF) yield: (**a**) temperature; (**b**) power; (**c**) time; (**d**) liquid-solid ratio.

The influence of temperature on the yield of extracted flavonoids was carried out at 40, 45, 50, 55, and 60 °C (Figure 1a). Concurrently, the following experimental conditions were used: time 30 min, power 300 W, and liquid-solid ratio 30 mL/g. The flavonoid yield initially increased and then decreased with increasing time. This observation might be attributed to hot spots that caused a decline in the solvent viscosity, and the acceleration of molecular movement with increasing temperature; additionally, a higher temperature could cause sensitive flavonoids to be degraded [17]. Therefore, 50 °C was considered as the optimal temperature.

#### 2.1.2. Effect of Ultrasound Power

The effect of ultrasound power on the flavonoid yield was investigated at 180, 210, 240, 270, and 300 W, while other variables were 50 °C, 30 min, and 30 mL/g (Figure 1b). When the ultrasound power ranged from 180 to 270 W, the extraction yield increased from 71.52 to 79.03 mg/g. Obviously, ultrasound power was critical in increasing the flavonoid yield. The higher the ultrasound power, the more thoroughly the plant tissues and cell walls were broken due to the effects of cavitations generated by micro-jets [18]. The results indicated that 270 W was the optimal power.

#### 2.1.3. Effect of Extraction Time

The influence of extraction time on the flavonoid yield was studied at 30, 40, 50, 60, and 70 min, with power at 300 W, temperature at 50 °C, and liquid-solid ratio of 30 mL/g (Figure 1c). The extract yield was obviously increased by extending the extraction time, when time varied from 30 to 50 min. The maximum yield (76.79 mg/g) was obtained at 50 min, but declined when time varied from 50 to 60 min. This result might be due to the bioactive mass transferred from the sample matrix into the solvent by diffusion [19]. Thus, 50 min was selected as the optimal time.

#### 2.1.4. Effect of Liquid-Solid Ratio

**Figure 2:**
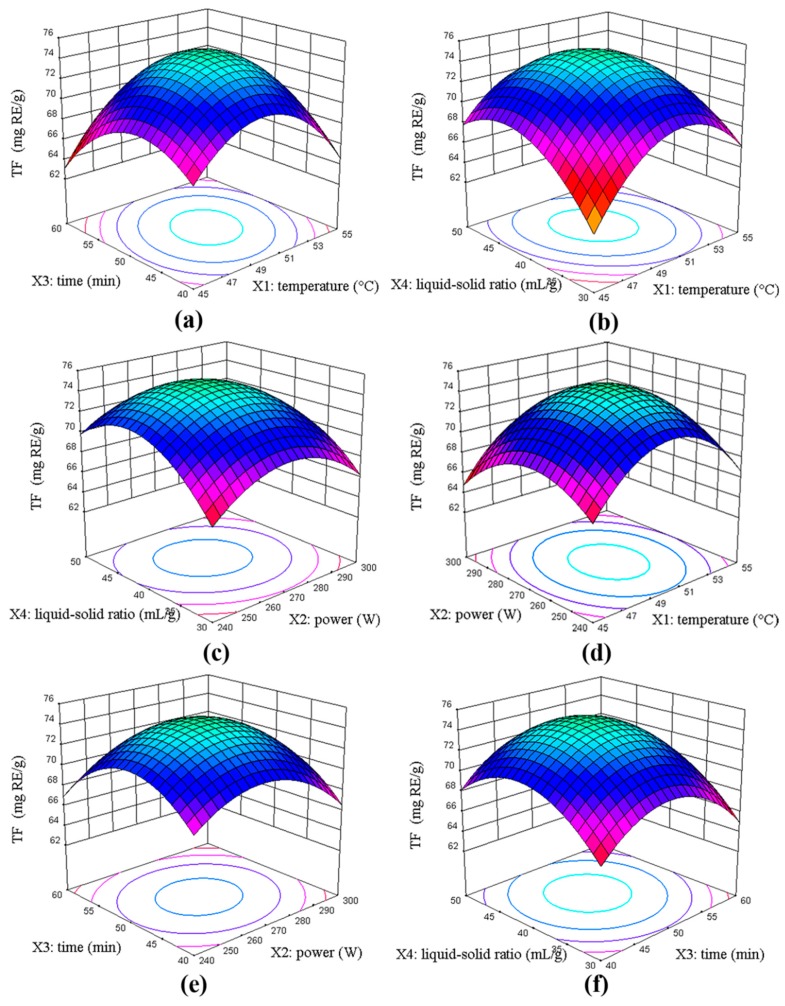
Response surface plots of the four factors interaction on the total flavonoids (TF) yield. (**a**) temperature and time (power 270 W, liquid-solid ratio 40 mL/g); (**b**)temperature and liquid-solid ratio (power 270 W, time 50 min); (**c**) power and liquid-solid ratio (time 50 min, temperature 50 °C); (**d**) power and temperature (time 50 min, liquid-solid ratio 40 mL/g); (**e**) power and time (temperature 50 °C, liquid-solid ratio 40 mL/g); (**f**) time and liquid-solid ratio (temperature 50 °C, power 270 W).

Liquid-solid ratio, an important extraction parameter, was set at 20, 30, 40, 50, and 60 mL/g, and the other parameters were as follows: power 300 W, temperatures 50 °C, and time 30 min (Figure 2d). When the liquid-solid ratio increased from 20 to 40 mL/g, the extraction yield rapidly increased and reached its maximum yield (74.31 mg/g) at 40 mL/g. These phenomena implied that the rate of diffusion was directly proportional to the concentration gradient, which increases at lower solid-liquid ratios [20]. From an economical point of view, the extraction process of flavonoids would be more feasible and efficient in potential applications that consume less solvent. Thus, 40 mL/g was selected as the optimum liquid-solid ratio.

### 2.2. Response Surface Optimization of Flavonoids

#### 2.2.1. Analysis of Extraction Model

The extraction yield of flavonoids at different experimental combinations is presented in Table 1. Four parameters of temperature (X_1_), power (X_2_), time (X_3_), and liquid-solid ratio (X_4_) were investigated. The regression model could be described by the following quadratic polynomial in terms of coded values:

Y = 42.02 + 0.40X_1_ − 0.56X_2_ − 0.38X_3_ + 1.22X_4_ + 0.28X_1_X_2_ + 1.49X_1_X_3_ − 1.33X_1_X_4_ +0.29X_2_X_3_ − 0.67X_2_X_4_ + 0.17X_3_X_4_ − 5.24X_1_^2^ − 3.53X_2_^2^ − 4.18X_3_^2^ − 3.83X_4_^2^ (1)

The ANOVA for the fitted quadratic model is presented in Table 2. The high *F*-value (285.81) and low *p*-value (<0.0001) suggested that the regression model was highly significant. The lack of fit of the *F*-value of 4.31 and the associated *p*-value of 0.0860 were not significant, indicating that the model equation was adequate for predicting the extraction yield of flavonoids. The high coefficient (R^2^) and high adjusted determination coefficient (R^2^_adj_) were 0.9965 and 0.9930, respectively, indicating a high correlation between the predicted and experimental values. A very low coefficient of variation (C.V.%) of 0.44 denoted that the experimental results were highly reliable. Furthermore, the *p*-values were used to check the significance of each coefficient, and a larger *F*-value and smaller *p*-value indicate a more significant coefficient [21]. As a result, the linear coefficients (X_1_, X_2_, X_3_, X_4_), interaction coefficients (X_1_X_3_, X_1_X_4_, X_2_X_4_), and quadratic terms (X_1_^2^, X_2_^2^, X_3_^2^, X_4_^2^) were significant (*p* < 0.05), whereas the other coefficients (X_1_X_2_, X_2_X_3_, X_3_X_4_) were insignificant (*p* > 0.05).

#### 2.2.2. Analysis of Response Surface

Three-dimensional (3D) response surfaces were provided as graphical representations of the regression equation (Figure 2). Figure 2a shows that the increase in both temperature and time (X_1_X_3_) accelerated the extraction of flavonoids at a liquid-solid ratio of 40 mL/g and power at 270 W. However, beyond 50.80 °C and 50 min, the extraction yield decreased. From Figure 2b, the extraction yield increased by simultaneously increasing the extraction temperature and liquid-solid ratio (X_1_X_4_) at time 50 min and liquid-solid ratio 40 mL/g. A higher yield of flavonoids obtained at 40 mL/g implied that the interaction effect of X_1_X_4_ had a more positive significant effect. Figure 2c describes the interaction effects of power and liquid-solid ratio (X_2_X_4_) at temperature 50 °C and time 50 min. The effect of the liquid-solid ratio on flavonoid yield displayed a linear increase, but power slightly increased. Therefore, from these response surfaces and ANOVA analyses (Table 2), it was evident that the liquid-solid ratio was the most significant factor affecting extraction yield, followed by ultrasound power, temperature, and time.

Figure 2d shows the effect between temperature and power (X_1_X_2_) on flavonoid yield when the extraction liquid-solid ratio and time were fixed. The mutual interactions of X_1_X_2_ were not significant. As shown in Figure 2e, the extraction power and time (X_2_X_3_) have a similar slight effect on flavonoid yield when the temperature and the liquid-solid ratio were fixed. From Figure 2f, the negative effect between extraction time and liquid-solid ratio (X_3_X_4_) when temperature and power were fixed might suggest that higher power and longer time will adversely affect the separation of the solid and liquid phases. Therefore, the response optimizer plots demonstrated that the interaction effects of X_1_X_2_, X_2_X_3_, and X_3_X_4_ were insignificant.

#### 2.2.3. Optimization of Extraction Conditions

The effectiveness of response surface modeling for UAE is reflected in Table 1. The predicted optimal extraction yield was 74.93 mg/g under the following conditions: 50.04 °C, 267.09 W, 49.55 min, and 41.65 mL/g, which was close to the experimental extraction yield (74.95 ± 0.13 mg/g) under actual operating conditions (50 °C, 270 W, 50 min, and 41 mL/g).

### 2.3. Analysis of Flavonoid Compositions

The content of total flavonoids from the aged and young leaves from April and July was detected under optimized conditions (Table 3). The results showed that the total flavonoid content in April was higher than that of July, and the highest content (74.81 mg RE/g dm) was measured in the aged leaves, followed by young leaves (58.17 mg RE/g dm). Thus, the effect of leaf age and seasons on total flavonoid content was significant, and was also observed in other work [22], where it was shown that there was a decrease in total flavonoids when the leaves were still growing.

**Figure 3:**
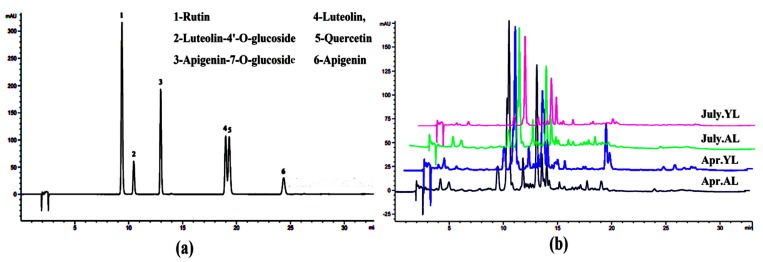
HPLC chromatograms of flavonoid compounds: (**a**) mixture standard; (**b**) samples from the aged and young leaves (AL and YL) in April and July.

The HPLC chromatogram of samples is given in Figure 3b. Six constituents were identified from the sample as rutin (1), luteolin-4’-*O*-glucoside (2), apigenin-7-*O*-glucoside (3), luteolin (4), quercetin (5), and apigenin (6) by matching their retention times against those of the standards (Figure 3a). It is worth mentioning that the retention times of luteolin and quercetin are close, which may be due to their very similar polarities; and this phenomenon has been noted in a previous work by Sánchezrabaneda et al. [23]. All identified flavonoids were quantified based on the calibration equations in Table 3. Compared with young leaves, the level of luteolin-4’-*O*-glucoside (31.02 mg/g dm) was higher in aged leaves (Table 3), whereas luteolin was lower; apigenin and quercetin could not be detected. Interestingly, the amounts of apigenin-7-*O*-glucoside and rutin increased in aged leaves. Our findings were in agreement with Abaza, et al. [24], who reported that the aged leaves contained higher levels of luteolin, while young leaves presented higher content of flavonoid aglycones. Perhaps this phenomenon of uneven distribution can be attributed to the conversion of flavonoid aglycones into glycones during flavonoid biosynthesis in the process of leaf growth [25,26].

### 2.4. Assay of Antioxidant Activity

#### 2.4.1. Reducing Power

**Figure 4:**
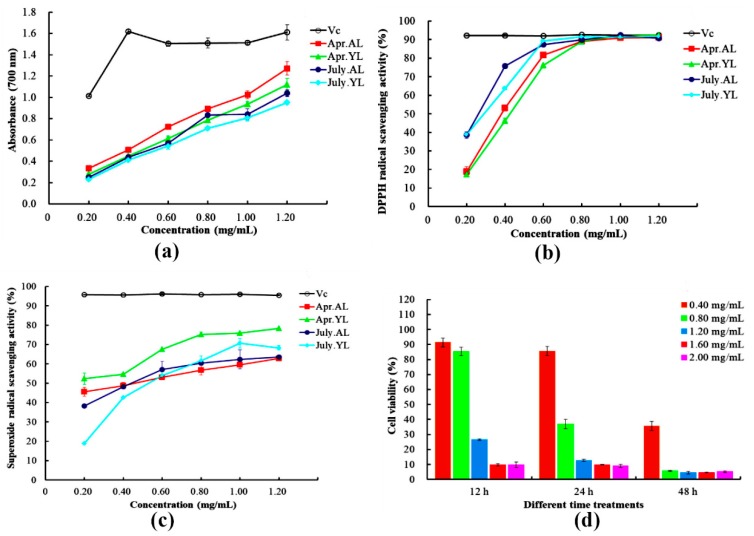
Antioxidant and anticancer activities of samples with different concentrations (mg of RE/mL of flavonoids extract). (**a**) Ruding power; (**b**) DPPH radical scavenging activity; (**c**) Superoxide radical scavenging activity; (**d**) Time dependent dose-response of HeLa cells determined with CCK-8.

Reducing power, which reflects the electron donation capacity, may serve as a significant antioxidant indicator [27]. Figure 4a shows that a dose-response relationship was observed in all of the samples. The absorbances of Apr.AL, Apr.YL, July.AL, and July.YL were 1.27, 1.12, 1.04, and 0.95 respectively under 1.20 mg/mL of flavonoids extract, whereas that of Vc was 1.61 mg/mL. Apr.AL displayed the highest reducing power, but a lower Vc (ascorbic acid) level. A similar result was found in a study by Nadeem, Ubaid, Qureshi, Munir and Mehmood [14], which demonstrated that the increase of flavonoids from juice blends could enhance their antioxidant activity. These results indicated that Apr.AL with higher reducing power might possess higher amounts of total flavonoid content [28].

#### 2.4.2. DPPH Radical Scavenging Activity

The DPPH assay is widely used to evaluate the free radical scavenging ability of antioxidants [29]. Figure 4b indicates the DPPH radical scavenging activity of samples at different concentration treatments. At 1.00 mg/mL of flavonoids extract, the radical scavenging capacities of Apr.AL, Apr.YL, July.AL, and July.YL achieved 91.01, 92.16, 92.39, and 91.38%, respectively, which were nearly equal to that of Vc (92.47%). The half-effective concentration (EC_50_, mg/mL) increased as follows: July.AL (0.24) < July.YL (0.26) < Apr.YL (0.33) < Apr.AL (0.36). The higher activity from July.AL and July.YL might be related to the capability of potential hydrogen-donating antioxidants to convert the stable radical DPPH into its reduced form DPPH-H [30]. It might be considered that other factors such as the proportion of flavonoid compounds and the position of hydroxyl groups influenced the DPPH scavenging capacity [31], but the specific mechanisms acting upon antioxidant activity remain to be investigated further.

#### 2.4.3. Superoxide Radical Scavenging Activity

Superoxide radicals can quickly react with other reactive oxygen species and form stronger free radicals [32]. As shown in Figure 4c, at the extract of 1.20 mg/mL, the scavenging effectiveness of Apr.AL, Apr.YL, July.AL, and July.YL were 62.85, 78.27, 63.59, and 68.32%, respectively, but the antioxidant potential of these samples was inferior to that of Vc (95.49%). The order of EC_50_ values (mg/mL) was Apr.YL (0.21) < Apr.AL (0.38) < July.AL (0.43) < July.YL (0.55), which suggests that Apr.YL was the best, a fact that might be attributed to the strong antioxidant activities of quercetin and luteolin [33].

### 2.5. Assay of Cell Viability

The sample of aged leaves from April was selected as the experimental material according to the determination of total flavonoid content. As shown in Figure 4d, a dose-dependent increase of cell viability was observed when HeLa cells were treated with 0.40–2.00 mg/mL of the extracts. At 12 h, the cell viability was 91.38, 85.26, 26.54, 9.66, and 9.79% at different concentrations, which indicated that the higher concentrations led to lower viability of HeLa cell. Similar dose-dependent results appeared at 24 h, and were more obvious at 48 h.

The order of EC_50_ (mg/mL) was 48 h (0.26) < 24 h (0.69) < 12 h (1.05), implying that there was more than a 2-fold dose increase at 24 h compared to 48 h, which was similar to the time-response by Quirantespiné, et al. [34]. Accordingly, after treating with 1.60 mg/mL, cell viability was 9.66, 9.83, and 4.59% at 12, 24, and 48 h, respectively. This finding denoted that continuous prolonged time exposures (24 or 48 h) of the HeLa cells with concentration exceeding 1.60 mg/mL resulted in significantly decreasing cell viability. It might be linked to the anti-inflammatory and anticancer properties of apigenin-7-*O*-glucoside and luteolin in olive leaves, despite their relatively low concentrations [35], and other compounds might also be involved in this effect, which will be further validated by future in vivo experiments.

## 3. Material and Methods

### 3.1. Materials and Chemicals

The Manzanillo variety of olive trees was cultivated in the commercial olive orchards of Xichang, Sichuan. Olive leaves were collected in April and July, 2017. The leaves were washed and dried at 45 °C, and then ground into powder by a high speed mill (FW177, Taisite Instrument Co., Ltd., Tinjin, China). The powder was stored at −20 °C.

Phenazine methosulfate (PMS), 1,1-diphenyl-2-picrylhydrazyl (DPPH), nicotinamide adenine dinucleotide (NADH), and nitroblue tetrazolium (NBT) were obtained from Sigma Chemical Co. (St. Louis, MO, USA). Fetal bovine serum (FBS) and penicillin–streptomycin were from Gibco/BRL (Burlington, Canada). HPLC-grade apigenin-7-*O*-β-d-glucosidase, apigenin, quercetin, rutin, luteolin, and luteolin-4’-*O*-glucoside were purchased from the Chengdu Must Biotechnology Co., LTD (Chengdu, China); acetonitrile was purchased from Acros Organics (Geel, Belgium). All other reagents were analytical grade and obtained from Chengdu Kelong Chemical Factory (Chengdu, China).

### 3.2. Ultrasound-Assisted Extraction (UAE)

Dried powder (1 g) of aged leaves from April was mixed with methanol in 100-mL tubes. The UAE parameters were liquid-solid ratio (20, 30, 40, 50, and 60 mL/g), power (180, 210, 240, 270, and 300 W), time (30, 40, 50, 60, and 70 min) and temperature (40, 45, 50, 55, and 60 °C), of which single factor experiments were performed, respectively. These samples were subjected to ultrasound using an ultrasonic device (KQ300DV, 40 kHz, Kunshan Ultrasonic Instrument Co., Jiangsu, China). After extraction, the supernatant was collected by centrifugation at 5000 rpm for 10 min and stored at −20 °C for further analysis.

### 3.3. Response Surface Methodology (RSM) Design

Based on the single-factor results, RSM was used to optimize the UAE conditions for extraction of flavonoids from olive leaves. A Box-Behnken design (BBD) with four factors (X_1_, temperature; X_2_, power; X_3_, time; X_4_, liquid-solid ratio) and three levels (−1, 0, 1) was performed. The extraction yield of flavonoids was taken as the response. The coded and actual levels of the four factors are presented in Table 1. A quadratic model was fitted to correlate the relationship between the independent variables and the response in order to predict the optimal conditions:

(2) $$Y=b_{0}+\sum_{i=1}^{4}b_{i}x_{i}+\sum_{i=1}^{4}b_{ii}x_{i}^{2}+\sum_{i<j=2}^{4}b_{ij}x_{i}x_{j}$$

where Y is the predicted extraction yield; *b*_0_ denotes a constant; *b_i_*, *b_ii_*, and *b_ij_* denote coefficients of the linear, quadratic, and interaction terms, respectively; and *x_i_* and *x_j_* denote independent variables.

### 3.4. Determination of Total Flavonoids

Quantitation of flavonoids in the extract solution was performed using the aluminum chloride (AlCl_3_) method [36] with some modifications. Briefly, 2 mL supernatants were mixed with 4 mL 1% AlCl_3_, and then was diluted to 25 mL with 30% methanol solution. Then, the absorbance was measured at 415 nm with a microplate reader (Spectramax M2, USA). The amount of total flavonoids is displayed as rutin equivalents through a standard calibration curve (0.2–1.0 mg/mL), and the outcome of total flavonoids contents was expressed as mg of routine equivalents (RE) per g of dry matter (dm). The total flavonoids yield was measured using the following equation: Yield (mg RE/g) = the mass of extracted flavonoids (mg)/the mass of dried sample (g).

### 3.5. HPLC Analysis

The chromatographic analysis was performed using an Agilent 1260 HPLC (Agilent Technologies, Santa Clara, CA, USA) coupled with a UV-Vis DAD detector at 350 nm and a C18 reversed-phase column (5.0 µm, 150 mm × 4.6 mm). Aqueous phosphoric acid (0.2%, *v*/*v*) and acetonitrile were used as mobile phase A and mobile phase B, respectively. The gradient program was as follows: 10–16% B (0–3 min), 16–30% B (3–20 min), 30–40% B (20–25 min), 40–16% B (25–30 min), and 16–10% B (30–33 min). The flow rate was 0.8 mL/min. The column temperature was maintained at 30 °C, and the injection volume was 10 µL. The concentration of rutin, luteolin-4’-*O*-glucoside, apigenin-7-*O*-glucoside, luteolin, quercetin, and apigenin were determined using the standard calibration curve, and the results are presented as milligrams per gram of dry matter (dm).

### 3.6. Antioxidant Activity Assays

#### 3.6.1. Reducing Power

The reducing power was determined by the Fe^3+^ reduction method [37]. For this procedure, 0.2 mL sample solution (flavonoids extract) was mixed with 0.5 mL phosphate buffer (0.2 M, pH 6.6) and 0.5 mL 1% potassium ferricyanide. The mixture was incubated at 50 °C for 20 min in a water bath, and then 0.5 mL 10% trichloroacetic acid was added, and the solution was centrifuged (3000 rpm, 10 min). Next, 0.5 mL supernatant was mixed with 0.5 mL distilled water, and then reacted with 0.1 mL 0.1% ferric chloride. The absorbance of the mixture was measured at 700 nm (Spectramax M2). Distilled water and ascorbic acid (Vc) served as the blank and positive control, respectively.

#### 3.6.2. DPPH Radical Scavenging Activity

The antioxidant capacity was measured using the DPPH assay, as described by Brand-Williams, Cuvelier and Berset [29], with minor modifications. Briefly, DPPH (0.4 mM) and different solutions of flavonoids extract (0.2, 0.4, 0.6 0.8, 1.0, and 1.2 mg/mL) were prepared with methanol. Then, 65-µL samples were mixed with 135 µL DPPH solution at 37 °C for 10 min in the dark. Methanol and Vc served as the blank and positive control, respectively. Absorbance at 517 nm was measured. The scavenging activity was calculated with the following equation: DPPH radical scavenging activity (%) = (A_control_ − A_sample_)/A_control_) × 100. The EC_50_ value is the concentration of sample required for 50% scavenging of the DPPH free radical, which was determined from the plot between %inhibition and concentration; from this, the average EC_50_ value was calculated.

#### 3.6.3. Superoxide Radical-Scavenging Activity

The ability of olive leaf to scavenge superoxide, an oxygen free radical, was determined based on the protocol by Jia et al. [38] with minor modifications. Briefly, 0.2-mL samples (0.2–2.0 mg/mL) were mixed with 0.2 mL NBT (0.08 mM), 0.4 mL NADH (0.25 mM), and 0.2 mL PMS (0.06 mM) at 25 °C for 25 min. The absorbance at 560 nm was recorded. The negative control was distilled water. The scavenging effect was calculated with the following equation: Superoxide radical scavenging activity (%) = (A_control_ − A_sample_)/A_control_) × 100.

### 3.7. Anticancer Activity Assays

#### 3.7.1. Cell Culture

HeLa cells (human cervical cancer cells) were obtained from the Biotechnology Center of Sichuan University (Sichuan, China). Cells were cultured in Dulbecco’s modified Eagle’s medium (DMEM) containing 10% fetal bovine serum (FBS) and 1% penicillin-streptomycin in a 5% CO_2_ atmosphere at 37 °C in a humidified incubator.

#### 3.7.2. Cell Viability

Cell Counting Kit-8 (CCK-8; Dojindo, Kumamoto, Japan) assays were performed to detect the cell viability according to the manufacturer’s instruction. Next, the sample was dissolved in DMEM medium, and the cells were treated with different sample concentrations (0, 0.4, 0.8, 1.2, 1.6, and 2.0 mg RE/mL of flavonoids extract, respectively) for different periods of time (12, 24, and 48 h), and then DMEM medium was completely replaced by 100 µL fresh growth medium. Next, 10 µL CCK-8 was added to cell wells and incubated 1 h. Finally, viable cells were counted at 450 nm (Spectramax M2). Cell viability was determined according to the following equation: Cell viability (%) = (A_test_ − A_blank_)/(A_control_ − A_blank_) × 100, where A_test_ was the absorbance of a well with cells, CCK-8 solution, and sample solution; A_blank_ was the absorbance of a well with medium and CCK-8 solution, without cells; and A_control_ was the absorbance of a well with cells and CCK-8 solution, without a sample solution.

### 3.8. Statistical Analysis

The experimental results of the response surface design were analyzed using Design-Expert 10 software (trial version, State-Ease Inc., Minneapolis, MN, USA). All experiments were carried out in triplicate, and the data are reported as the mean ± standard deviation (SD). Statistical analysis was determined using a one-way analysis of variance (ANOVA, SPSS version 17 Inc., Chicago, IL, USA). *p* value < 0.05 was regarded as significant.

## 4. Conclusions

In the present study, RSM was successfully applied for the optimization of the UAE process to extract flavonoids from olive leaves. The maximum yield of flavonoids was 74.95 mg RE/g, when the optimal extraction conditions were temperature 50 °C, power 270 W, time 50 min, and liquid-solid ratio 41 mL/g. A significant difference of the total flavonoid content was observed between young and aged leaves from April and July, and six main constituents of flavonoids were quantified by HPLC. Among these compounds, luteolin-4’-*O*-glucoside was the most abundant compound, and apigenin-7-*O*-glucoside and rutin followed, but the levels of luteolin, apigenin, and quercetin were very low. Moreover, all of the samples exhibited good DPPH and superoxide radical scavenging activity, and reducing power. Furthermore, when HeLa cells were treated with various concentrations (mg of RE/mL of flavonoids solution) of bioflavonoids at different times, anticancer activity was exhibited in a concentration- and time-dependent manner, and stronger cell viability was presented at 24 h and 1.60 mg/mL. In summary, the flavonoids from Manzanillo olive leaves have great potential medicinal value, and should be comprehensively utilized as an antioxidant resource.

## Figures and Tables

**Table 1:** BBD design with the experimental and predicted values for extraction yield.

Run	Extraction Variables				Extraction Yield of Flavonoids (mg RE/g)
	X_1_ Temperature (°C)	X_2_ Power (W)	X_3_ Time (min)	X_4_ Liquid-Solid Ratio (mL/g)	
1	−1(45)	−1(240)	0(50)	0(40)	66.12
2	0(50)	−1(240)	0(50)	1(50)	69.84
3	−1(45)	0(270)	−1(40)	0(40)	66.72
4	0(50)	1(300)	0(50)	−1(30)	66.23
5	1(55)	0(270)	1(60)	0(40)	66.87
6	−1(45)	0(270)	0(50)	1(50)	68.26
7	1(55)	0(270)	0(50)	−1(30)	66.16
8	0(50)	−1(240)	−1(40)	0(40)	68.77
9	0(50)	0(270)	−1(40)	−1(30)	66.15
10	0(50)	0(270)	1(60)	1(50)	67.52
11	−1(45)	0(270)	1(60)	0(40)	62.98
12	−1(45)	1(300)	0(50)	0(40)	64.89
13	0(50)	0(270)	−1(40)	1(50)	67.84
14	0(50)	0(270)	0(50)	0(40)	74.91
15	0(50)	1(300)	1(60)	0(40)	66.30
16	0(50)	−1(240)	1(60)	0(40)	67.31
17	1(55)	1(300)	0(50)	0(40)	66.24
18	0(50)	0(270)	0(50)	0(40)	74.90
19	0(50)	0(270)	1(60)	−1(30)	65.16
20	1(55)	0(270)	0(50)	1(50)	66.30
21	1(55)	−1(240)	0(50)	0(40)	66.37
22	0(50)	1(300)	0(50)	1(50)	67.40
23	0(50)	0(270)	0(50)	0(40)	74.57
24	1(55)	0(270)	−1(60)	0(40)	64.67
25	0(50)	−1(240)	0(50)	−1(30)	66.00
26	0(50)	0(270)	0(50)	0(40)	74.91
27	0(50)	1(300)	−1(40)	0(40)	66.60
28	0(50)	0(270)	0(50)	0(40)	74.67
29	−1(45)	0(270)	0(50)	−1(30)	62.82
Predicted	50.10	267.32	49.62	41.64	74.93
Experimental	50.00	270.00	50.00	41.00	74.95

**Table 2:** ANOVA for response surface quadratic model analysis of extraction yield.

Source	Sum of Squares	df	Mean Square	*F*-value	*p*-Value
Model	348.72	14	24.91	285.81	0.0001 ^***^
X_1_	1.94	1	1.94	22.21	0.0003 ^***^
X_2_	3.8	1	3.8	43.57	0.0001 ^***^
X_3_	1.77	1	1.77	20.32	0.0005 ^***^
X_4_	17.86	1	17.86	204.94	0.0001 ^***^
X_1_X_2_	0.3	1	0.3	3.47	0.0836 ^ns^
X_1_X_3_	8.82	1	8.82	101.21	0.0001 ^***^
X_1_X_4_	7.02	1	7.02	80.58	0.0001 ^***^
X_2_X_3_	0.34	1	0.34	3.86	0.0696 ^ns^
X_2_X_4_	1.78	1	1.78	20.45	0.0005 ^***^
X_3_X_4_	0.11	1	0.11	1.29	0.2755 ^ns^
X_1_^2^	178.28	1	178.28	2045.69	0.0001 ^***^
X_2_^2^	81.01	1	81.01	929.49	0.0001 ^***^
X_3_^2^	113.41	1	113.41	1301.31	0.0001 ^***^
X_4_^2^	95.28	1	95.28	1093.29	0.0001 ^***^
Lack of Fit	1.12	10	0.11	4.31	0.0860 ^ns^
Residual	1.22	14	0.087	-	-
Pure Error	0.1	4	0.026	-	-
Cor Total	349.94	28	-	-	-
R^2^	0.9965	-	-	-	-
R^2^_adj_	0.9930	-	-	-	-
C.V.%	0.44	-	-	-	-

*** Highly significant (*p* < 0.001); ns, not significant (*p* > 0.05). (X_1_) extraction temperature (°C); (X_2_) extraction power (W); (X_3_) extraction time (min); (X_4_) liquid-solid ratio (mL/g).

**Table 3:** Compounds (CP), calibration equations (CE), correlation coefficients (CC), and quantification of flavonoid compounds from the aged and young leaves (AL and YL) in April and July *.

CP	CE	CC	April		July	
			AL	YL	AL	YL
Total flavonoids content	y=0.1793x − 0.0012	0.9993	74.81 ± 0.91a	58.17 ± 2.12b	48.29 ± 1.41c	34.06 ± 4.02 d
Rutin	y =1915.3x + 2.6063	0.9988	0.85 ± 0.02a	0.59 ± 0.01c	0.68 ± 0.01b	0.43 ± 0.01d
Luteolin-4’-*O*-glucoside	y =256.15x + 7.0455	0.9981	31.02 ± 0.48a	19.16 ± 0.37c	24.20 ± 0.36b	23.15 ± 0.85b
Apigenin-7-*O*-glucoside	y =2745.8x + 0.1299	1.0000	2.06 ± 0.04a	1.00 ± 0.02d	1.53 ± 0.01b	1.11 ± 0.01c
Luteolin	y =3157.00x − 5.0318	0.9997	0.16 ± 0.001b	0.60 ± 0.006a	0.07 ± 0.0010d	0.13 ± 0.004c
Quercetin	y = 4910.80x − 3.6071	0.9989	nd	0.0021 ± 0.00004	nd	0.0008 ± 0.00004
Apigenin	y = 378228x − 1.0121	0.9991	nd	0.038 ± 0.0005	nd	nd

* Values are presented as mean ± standard deviation; Total flavonoids content is expressed as mg RE/g dm (mg of routine equivalent/g of dry matter); and six compouns are expressed as mg/g dm (mg of analyte/g of dry matter); the date marked by different letters in a row indicated significant different (*p* < 0.05); nd, not detected. y: peak area; x: concentration as mg/mL.
